# Idiosyncratic recognition of UUG/UUA codons by modified nucleoside 5-taurinomethyluridine, τm^5^U present at ‘wobble’ position in anticodon loop of tRNA^Leu^: A molecular modeling approach

**DOI:** 10.1371/journal.pone.0176756

**Published:** 2017-04-28

**Authors:** Asmita S. Kamble, Prayagraj M. Fandilolu, Susmit B. Sambhare, Kailas D. Sonawane

**Affiliations:** 1 Structural Bioinformatics Unit, Department of Biochemistry, Shivaji University, Kolhapur, (M.S.), India; 2 Department of Microbiology, Shivaji University, Kolhapur, (M.S.), India; Tel Aviv University, ISRAEL

## Abstract

Lack of naturally occurring modified nucleoside 5-taurinomethyluridine (τm^5^U) at the ‘wobble’ 34^th^ position in tRNA^Leu^ causes mitochondrial myopathy, encephalopathy, lactic acidosis and stroke-like episodes (MELAS). The τm^5^U_34_ specifically recognizes UUG and UUA codons. Structural consequences of τm^5^U_34_ to read cognate codons have not been studied so far in detail at the atomic level. Hence, 50ns multiple molecular dynamics (MD) simulations of various anticodon stem loop (ASL) models of tRNA^Leu^ in presence and absence of τm^5^U_34_ along with UUG and UUA codons were performed to explore the dynamic behaviour of τm^5^U_34_ during codon recognition process. The MD simulation results revealed that τm^5^U_34_ recognizes G/A ending codons by ‘wobble’ as well as a novel ‘single’ hydrogen bonding interactions. RMSD and RMSF values indicate the comparative stability of the ASL models containing τm^5^U_34_ modification over the other models, lacking τm^5^U_34_. Another MD simulation study of 55S mammalian mitochondrial rRNA with tRNA^Leu^ showed crucial interactions between the A-site residues, A918, A919, G256 and codon-anticodon bases. Thus, these results could improve our understanding about the decoding efficiency of human mt tRNA^Leu^ with τm^5^U_34_ to recognize UUG and UUA codons.

## Introduction

Post-transcriptionally modified nucleosides are indispensable for conformational dynamics and contribute to the structure of anticodon loop domain of several tRNAs. The recognition as well as binding of tRNA anticodon to cognate codons comes precisely in the ordered structure, by means of a reduced entropic penalty to the ribosome [1]. Hence, codon-anticodon base pairing is essential for proficient translation process to mould a positive conformation by means of the ribosome [2–5]. Transfer RNA modifications at ‘wobble’ 34^th^ position are involved in codon–anticodon recognition, while a conserved purine at 37^th^ position helps to prevent extended Watson-Crick base pairing [6]. Similarly, a conserved pyrimidine at position 32 and a persistent U at 33^rd^ position play critical roles during codon-anticodon recognition [7]. The wobble base pair G.U is an ultimate element of RNA secondary structure specifically existing in almost every single class of RNA [8]. Moreover, the G.U wobble base pair has exclusive structural, chemical, dynamic and ligand binding properties [8]. The base pairing between G.U would be capable of forming two hydrogen bonds by interacting through the matching face of the base involved in Watson-Crick pairing [8].

Accurate functioning of tRNA necessitates a canonical three dimensional anticodon loop structure including U-turn motif characterized by various hydrogen bonding interactions such as N(1)_31_…HN(4)_39_, PO(1)… HN(3)_U33_ of 36^th^ nucleotide and N(7)_A35_…HO2ʹ_U33_ [7, 9–10]. Conserved elements of ASL comprise a purine at position 37 and non Watson-Crick iso-steric base pairs at 32^nd^ and 38^th^ positions [11]. This contributes to an additional sequence signature of anticodon loop apart from the conserved U-turn at 33^rd^ position and a frequently modified purine at 37^th^ position. Evidently, the role of tRNA sequence in codon recognition is not limited to the anticodon tri-nucleotide segment, but it correspondingly takes an account of other sequence elements present in the anticodon stem loop [12]. Several, molecular modeling studies revealed the significant role of ‘wobble’ base modification on codon-anticodon recognition [9, 13–15]. Recently, the role of wobble base pairing by a single ‘novel’ hydrogen bonding interaction has been discussed [9]. Similarly, novel base-pairing interactions have also been observed at the tRNA wobble position, which are crucial for accurate decoding of the genetic code [16].

The ribosomal A-site is a tRNA binding as well as mRNA decoding site. A-site residues of mammalian mitochondrial ribosome especially A918, A919 and G256 play an important role as a molecular switch to control the fidelity of mRNA decoding [17–18]. Consequently, codon recognition at A-site is a dynamic process which is precise and sensitive to factors like base modifications, divalent cations, temperature and is also controlled by interactions with the ribosome [6]. It has been previously reported that xm^5^U anticodons lean towards ‘G’ ending codons than ‘A’ ending codons [19]. Conformational changes of the ribosome and tRNA have been observed during A-site binding process [7]. Transfer RNAs are known to possess variety of post-transcriptionally modified nucleosides. Several modified nucleosides occurring at 34^th^ position interact with the 3^rd^ base of codon on mRNA, while the tRNA nucleosides, 35^th^ and 36^th^ interact with 2^nd^ and 1^st^ nucleosides of the codon respectively [7].

Post-transcriptionally modified nucleosides are the center of tRNA structure and function. They are position specific and contribute remarkably to maintain base stacking, modulate codon-anticodon binding, provide rigidity or flexibility to tRNA, enhance or restrict the scope of codon recognition and to facilitate translocation. Absence of post-transcriptional modifications deteriorates the binding of specific tRNAs to the ribosomal A or P sites [20]. Absence of a single modification can lead to consequences such as ribosomal frame shifting and loss of proper three dimensional fold of tRNA molecule. MELAS is a genetic disorder which results due to the absence of a crucial post transcriptional modification 5-taurinomethyluridine (τm^5^U) at the 34^th^ position in the anticodon loop of tRNA^Leu^. Structural significance of 5-taurinomethyluridine monophosphate (p-τm^5^U) has been studied earlier using computational methods [13]. However, its role in codon recognition has not yet been fully understood.

Thus, the aim of current study is to explore the structural consequences of the presence and absence of ‘wobble’ modified nucleoside τm^5^U_34_ on ASL of human mt tRNA^Leu^ during codon recognition process. The explorations have been done by using fully solvated molecular dynamics (MD) simulations of modified and unmodified ASL models along with UUG/UUA codons. Extending the study, another MD simulation was also performed on ASL of human mt tRNA^Leu^ and mRNA codon UUG with A-site residues of the 55S mammalian mitochondrial ribosome. Thus, these results could be helpful to understand the role of τm^5^U_34_ in proper codon recognition.

## Materials and methods

### Molecular dynamics simulations of ASL tRNA^Leu^ in presence and absence of τm^5^U_34_ with UUG/UUA codons

**Fig 1:**
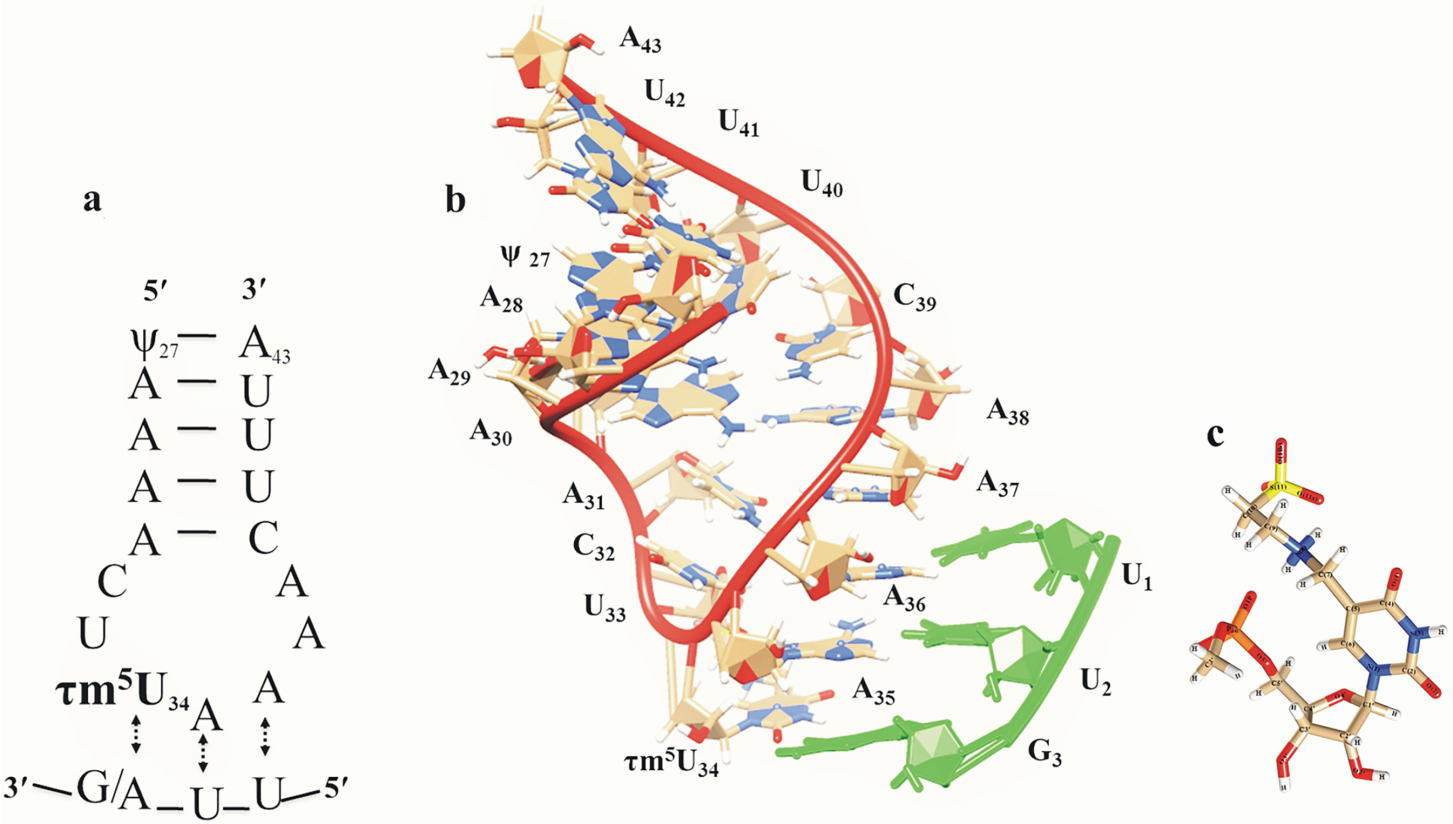
Model anticodon stem loop (ASL) with codon ‘UUG/A’ in a) the clover leaf model and b) three-dimensional structure of ASL of tRNA^Leu^ considered for MD simulation c) modification τm^5^U with nomenclature.

Models of anticodon stem loop (ASL) segments of tRNA^Leu^ along with UUG and UUA codons have been constructed for MD simulations as illustrated in Fig 1. For this, the ribose-phosphate backbone torsion angles of ASL tRNA^Leu^ were retained as per crystal structure (PDB ID:1EHZ) [21]. Nucleoside bases were modeled according to tRNA^Leu^ sequence [22] with the help of Tripos Sybyl 7.3 software [23]. Three-dimensional models of ‘UUG’ and ‘UUA’ codons were developed using Tripos Sybyl 7.3 and then physically docked to ASL tRNA^Leu^ model by maintaining proper hydrogen bonding interactions using Chimera [24]. Similar models of ASL tRNA^Leu^ with UUG/UUA codons were also developed by incorporating normal uridine (U) instead of τm^5^U at 34^th^ position. These models were used as a control for the MD simulation studies.

Molecular dynamics simulations were executed over the four models of ASL tRNA^Leu^; i) with τm^5^U_34_ modification: UUG codon, ii) without modification: UUG codon, iii) with τm^5^U_34_ modification: UUA codon and iv) without modification: UUA codon. All these models were solvated by 4326 TIP3P water molecules and neutralized by 19 Na^+^ ions in a rectilinear box having dimensions 65.28 x 50.37 x 55.97. MD trajectories were written at 2.0 fs time step using shake algorithm [25] for all hydrogen atoms by a 9.0 Å non-bonded cut off. The non-bonded pair list was updated at every 10 steps. The trajectories were calculated by keeping a constant temperature (300 K) and pressure (1atm) according to Berendsen coupling algorithm [26]. Simulations were executed under periodic boundary conditions using the Particle Mesh Ewald method to calculate long range interactions [27].

An equilibrium protocol, analogous to previous molecular dynamics simulation studies of modified nucleosides was applied [9, 28–29]. The equilibration convention comprised of 10,000 steps of steepest descent minimization followed by 500ps of MD at 300K has been applied to remove initial strain between water molecules and ASL models with codons. All ASL models were constrained, while water molecules and Na^+^ counter ions were allowed to move at 100 K (100ps), 200 K (100ps) and 300 K for 1.3ns. Accordingly, equilibration protocol was accomplished at 2.0ns. Equilibrated systems were then subjected to 5,000 steps of steepest descent minimization to eliminate hard contacts between water molecules and ASL models of tRNA^Leu^. During the production MD, no positional constraints were applied to the system and the temperature was gradually increased to 300 K by increment of 50 K per picosecond. Finally, all the four models were subjected to 50ns MD at 300 K temperature and constant pressure (1 atm) with fully solvated neutralized system using ff99bsc0 force field with the help of Amber 10 software [30]. The modified nucleoside parameters were adapted from ‘Modified Parameters Database server’ [31]. PTRAJ module of Amber 10 and Chimera software were used to analyze RMSD, RMSF, average and snapshot structures as well as trajectories generated during simulation [24, 32]. Molecular dynamics simulations were carried out using Amber 10 simulation suite on HP ProLiant-DL180G6 servers.

### MD simulation of 55S ribosomal A-site with ASL tRNA^Leu^ containing τm^5^U_34_ and UUG codon

The A-site residues, A918, A919, and G256 are known to play a crucial role in the translation process [17]. Initial coordinates for ASL tRNA^Leu^ with τm^5^U_34_ and UUG codon along with short patches (A916-C917-A918-A919-G920-U921 and C254-G255-G256-U257-C258) were extracted from 55S mammalian mitochondrial ribosome crystal structure as described in PDB ID 5AJ4 [17]. The nucleotide bases of ASL were edited as per the base composition of tRNA^Leu^ sequence [22], whereas mRNA codon bases were changed to U_1_-U_2_-G_3_ in the ribosomal complex. Fully solvated MD simulation was performed for 3.5ns using Amber 10. MD protocol was kept as discussed in the previous sub section of this study. The interactions of A-site residues of 55S mammalian mitochondrial ribosome with ASL of tRNA^Leu^ and codon UUG were analyzed using UCSF Chimera.

## Results

### Analysis of MD simulations of ASL tRNA^Leu^:UUG/UUA

#### Hydrogen bonding interactions of τm^5^U

**Fig 2:**
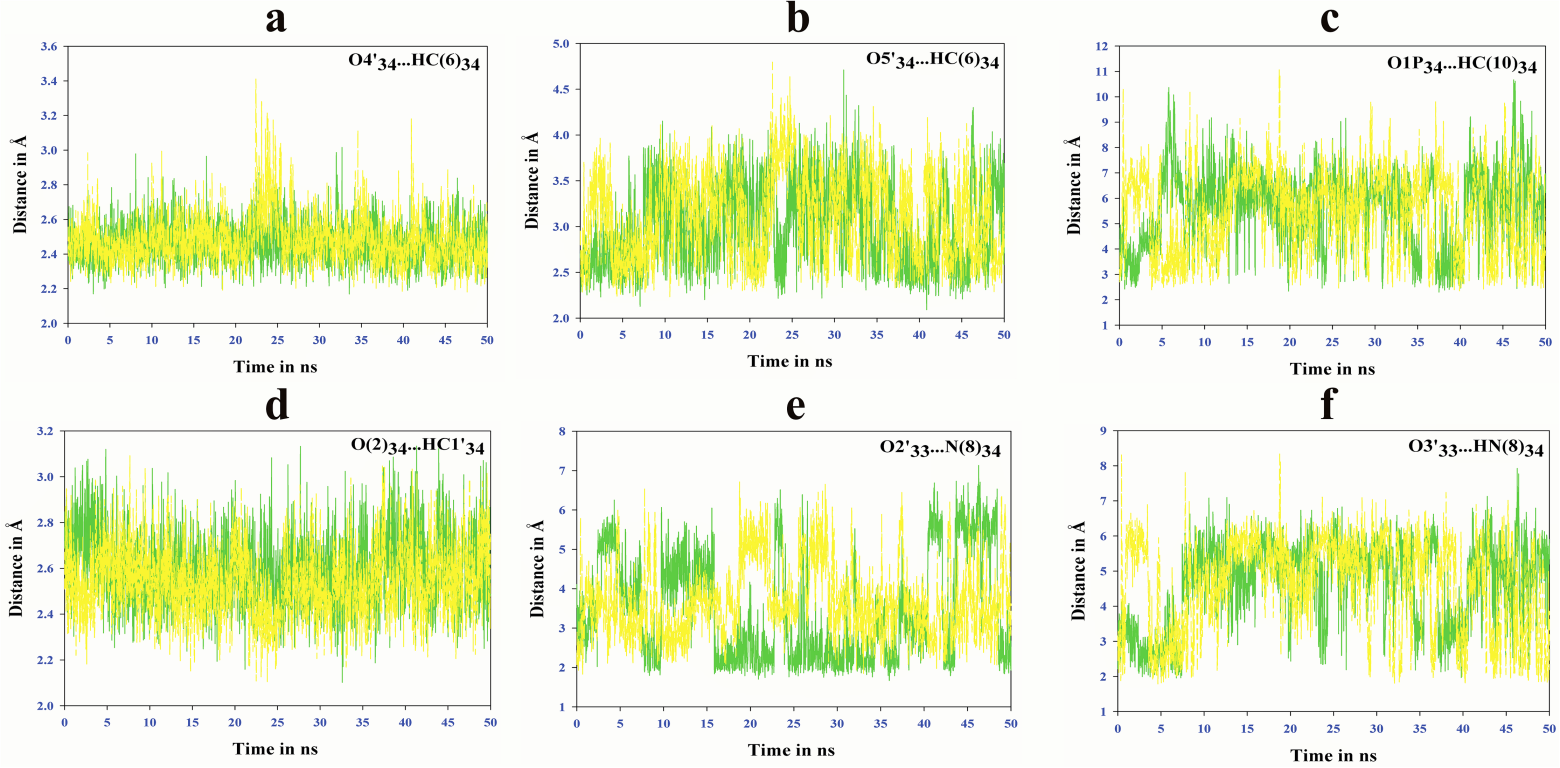
Hydrogen bonding interactions of τm^5^U.

Hydrogen bonds are crucial to bio-molecular functions. Multiple molecular dynamics simulations were carried out on ASL tRNA^Leu^ models (Fig 1), to assess conformational pliability of hypermodified nucleoside, τm^5^U_34_ along with UUG and UUA codons. MD simulation trajectories were analysed for hydrogen bonding interactions of τm^5^U_34_ within the ASL tRNA^Leu^. The hydrogen bonding interaction, O4'_34_…HC(6)_34_ (Fig 2A) between ribose ring and τm^5^U_34_ base has been found stable throughout MD simulation, whereas the interaction, O5'_34_…HC(6)_34_ (Fig 2B) was found disturbed. These interactions might help to maintain the ‘anti’ conformation of glycosyl torsion angle ‘χ’, as found in earlier studies [13–14]. The hydrogen bonding interaction, O1P_34_…HC(10)_34_ (Fig 2C) between phosphate backbone and τm^5^U_34_ side chain assists to retain ‘anti’ conformation of glycosyl torsion angle. The interaction, O(2)_34_…HC1'_34_ (Fig 2D) between τm^5^U_34_ base and ribose ring has been maintained during MD simulation, aiding to the stable behaviour of glycosyl torsion angle. In the context of ASL tRNA^Leu^, it has been observed that τm^5^U_34_ side chain also interacts with the ribose ring of U_33_ *viz*. O2'_33_…HN(8)_34_ (Fig 2E) and O3'_33_…HN(8)_34_ (Fig 2F), which could be helpful to maintain ‘anti’ conformation of glycosyl torsion angle as found in previous studies [13–14]. These hydrogen bonding interactions might be useful to retain the proper orientation of τm^5^U_34_ side chain. The anti-conformation of glycosyl torsion angle ‘χ’ allows the Watson-Crick base pairing sites O(4), N(3) and O(2) of τm^5^U_34_, free to interact with codons. The hydrogen bonding interactions and anti-conformation of glycosyl torsion angle ‘χ’ collectively result in increased integral stability rendering the atoms O(4), N(3) and O(2) of τm^5^U_34_ freely available for Watson-Crick base pairing.

#### Hydrogen bonding interactions within ASL tRNA^Leu^

**Fig 3:**
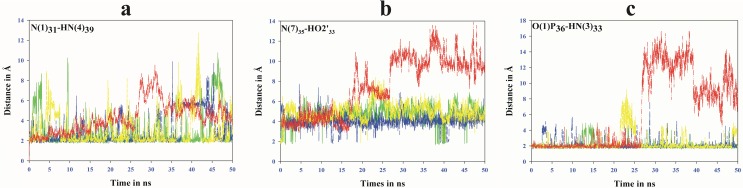
Hydrogen bonding interactions within ASL of tRNA^Leu^.

Hydrogen bonding interactions between τm^5^U_34_ side chain and other bases present in ASL tRNA^Leu^ are described in Fig 3A–3C. The interaction between N(1) of A_31_ and HN(4) of C_39_ (Fig 3A) maintains base stacking within the anticodon stem of tRNA^Leu^, similar to earlier report [9]. In the present study, this interaction was found stable in presence of τm^5^U_34_ modification, whereas it was disturbed in the absence of τm^5^U_34_ (Fig 3A). The U-turn feature described by hydrogen bonding interaction between N(7)_35_ and HO2'_33_ (Fig 3B) has been found maintained (Green color in Fig 3B) throughout 50ns MD simulation trajectory of ASL tRNA^Leu^ with τm^5^U_34_ modification. In contrast, ASL without τm^5^U_34_ shows highly distorted bonding between N(7)_35_ and HO2'_33_ (blue color in Fig 3B). Another interaction between O(1)P_36_ and N(3)_33_ (Fig 3C) also supports the U-turn feature as observed in previous study [7]. These hydrogen bonding interactions maintained internal base stacking to conserve the ‘U turn’ feature of ASL tRNA^Leu^.

#### Hydrogen bonding interactions between codon-anticodon base pair of UUG/UUA

**Fig 4:**
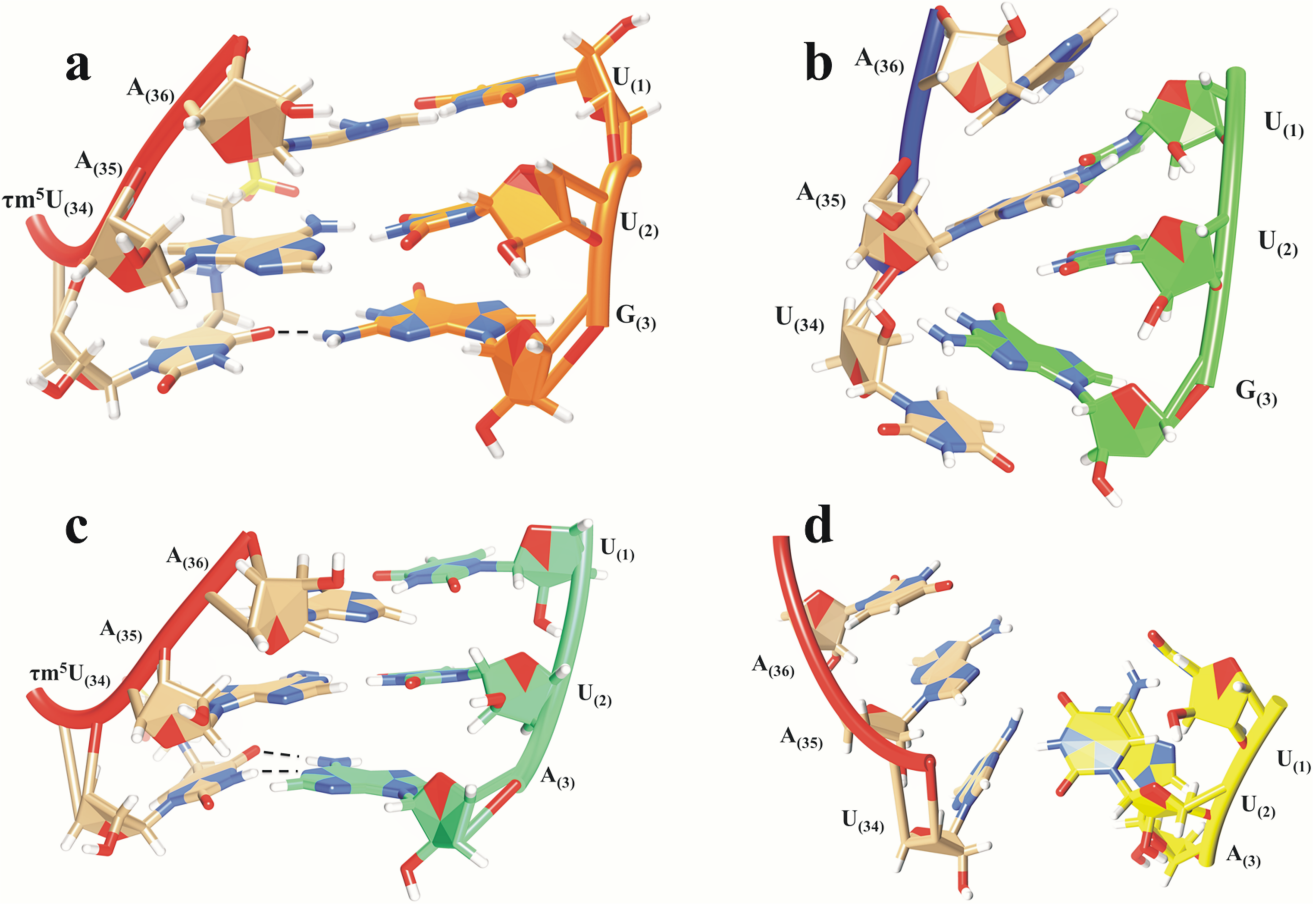
The 50ns snap shot structures showing hydrogen bonding and base stacking interactions between ASL and codon. a) model i) tRNA^Leu^ with modification τm^5^U:UUG codon b) model ii) tRNA^Leu^ without modification τm^5^U:UUG codon c) model iii) tRNA^Leu^ with modification τm^5^U:UUA codon d) model iv) tRNA^Leu^ without modification τm^5^U:UUA codon.

In order to view base stacking interactions among all bases in presence of codons, 50ns snapshots were analysed from all models of ASL tRNA^Leu^. In model i), ASL tRNA^Leu^ with τm^5^U_34_ modification and UUG codon, proper base stacking was observed as can be seen in Fig 4A. In this snapshot, τm^5^U_34_ plays a crucial role in base stacking to adhere to the third base of UUG codon. These results are in comparison with earlier report, suggesting that the xm^5^U anticodons lean towards G ending codons than A ending codons [19]. We have found a single base pairing recognition between O(4)_34_…HN(2)_G3_ (Fig 4A), similar to earlier findings [9, 33]. We did not observe proper base stacking interaction in model ii) i.e. without τm^5^U_34_ modification. The bases of UUG codon show distorted orientation resulting in lack of interactions between ASL and codons as can be seen in Fig 4B. Model iii), ASL tRNA^Leu^ with τm^5^U_34_ modification and UUA codon (Fig 4C) also shows proper base stacking interactions similar to model i) (Fig 4A). However, distorted stacking and base pairing interactions were observed in model iv), ASL tRNA^Leu^ without modification along with codon UUA (Fig 4D). The interactions observed in model i), τm^5^U_34_ and UUG codon are more stable as compared to other three models, indicating the importance of τm^5^U_34_ and its preference towards UUG codon than UUA.

#### Single and double hydrogen bonding interactions within the codon-anticodon bases

**Fig 5:**
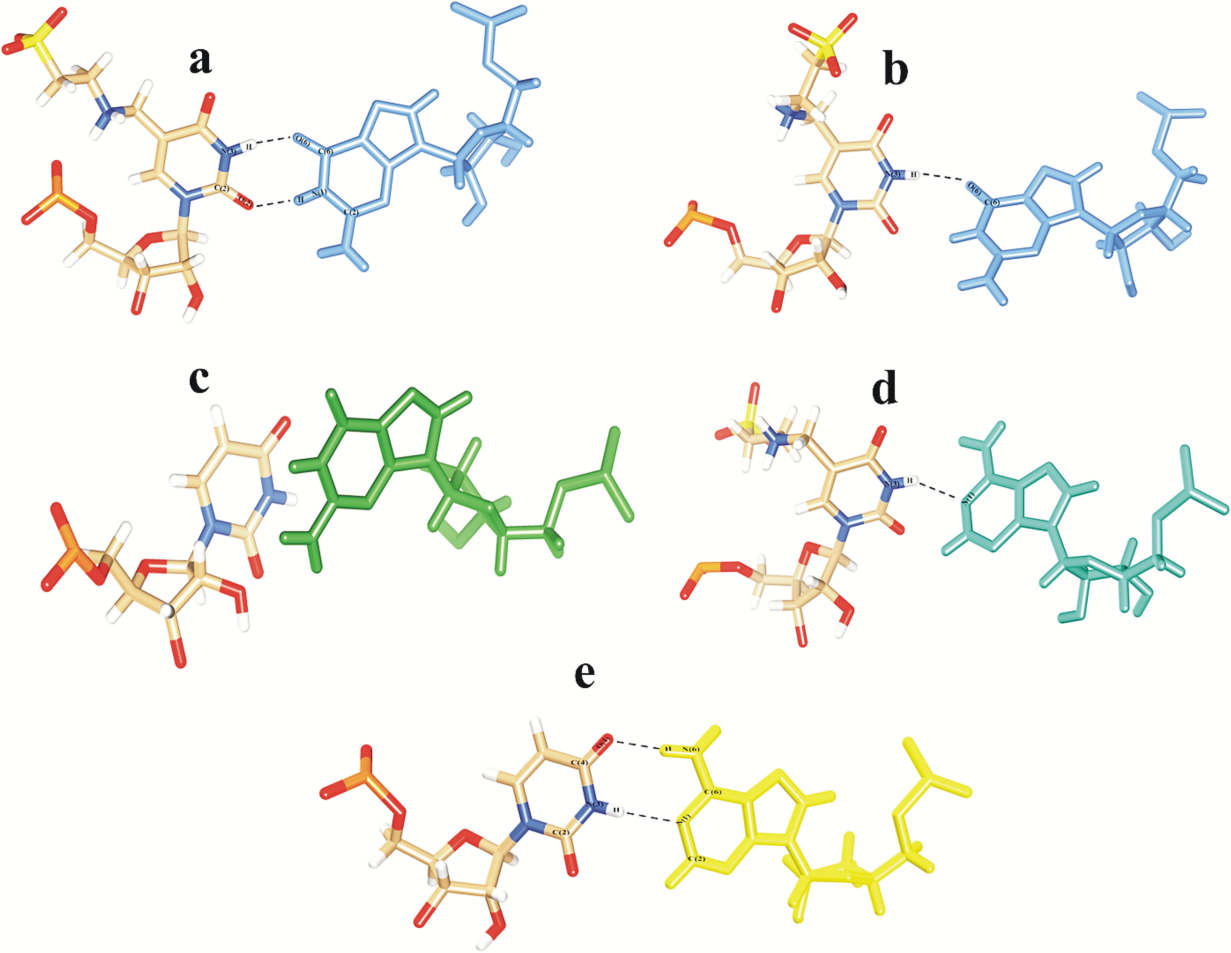
Hydrogen bonding interactions of a) τm^5^U_34_:G_3_ in tRNA^Leu^ showing recognition by double bond, b) τm^5^U_34_:G_3_ in tRNA^Leu^ showing single bond recognition, c) U_34_:G_3_ in tRNA^Leu^ showing no bonding interactions, d) τm^5^U_34_:A_3_ in tRNA^Leu^ showing single bond, e) U_34_:A_3_ in tRNA^Leu^ showing double bond, f) U_34_:A_3_ in tRNA^Leu^ showing bonding interactions.

The τm^5^U_34_ side chain of ASL tRNA^Leu^ interacts with G_3_ of UUG codon by ‘wobble’ hydrogen bonding between O(6)_G3_…HN(3)_34_ and O(2)_34_…HN(1)_G3_ (Fig 5A). However, this model is also stabilized by a single hydrogen bonded base pairing between O(6)_G3_…HN(3)_34_ (Fig 5B) similar to recent reports [9, 33]. In model ii), the unmodified uridine at 34^th^ position of ASL does not interact with G_3_ of UUG codon (Fig 5C). The interaction, N(1)_34_…HN(3)_A3_ (Fig 5D) between τm^5^U_34_ and A_3_ of UUA codon of model iii), also depicts a single hydrogen bond. Model iv) is stabilized by double hydrogen bonding interactions *viz*. O(4)_34_…HN(6)_A3_ and N(1)_A3_…HN(3)_34_ (Fig 5E). Such type of double hydrogen bonding interactions has also been reported in *E*. *coli* tRNA^Leu^ crystal structure [34].

#### RMSD of MD simulations

**Fig 6:**
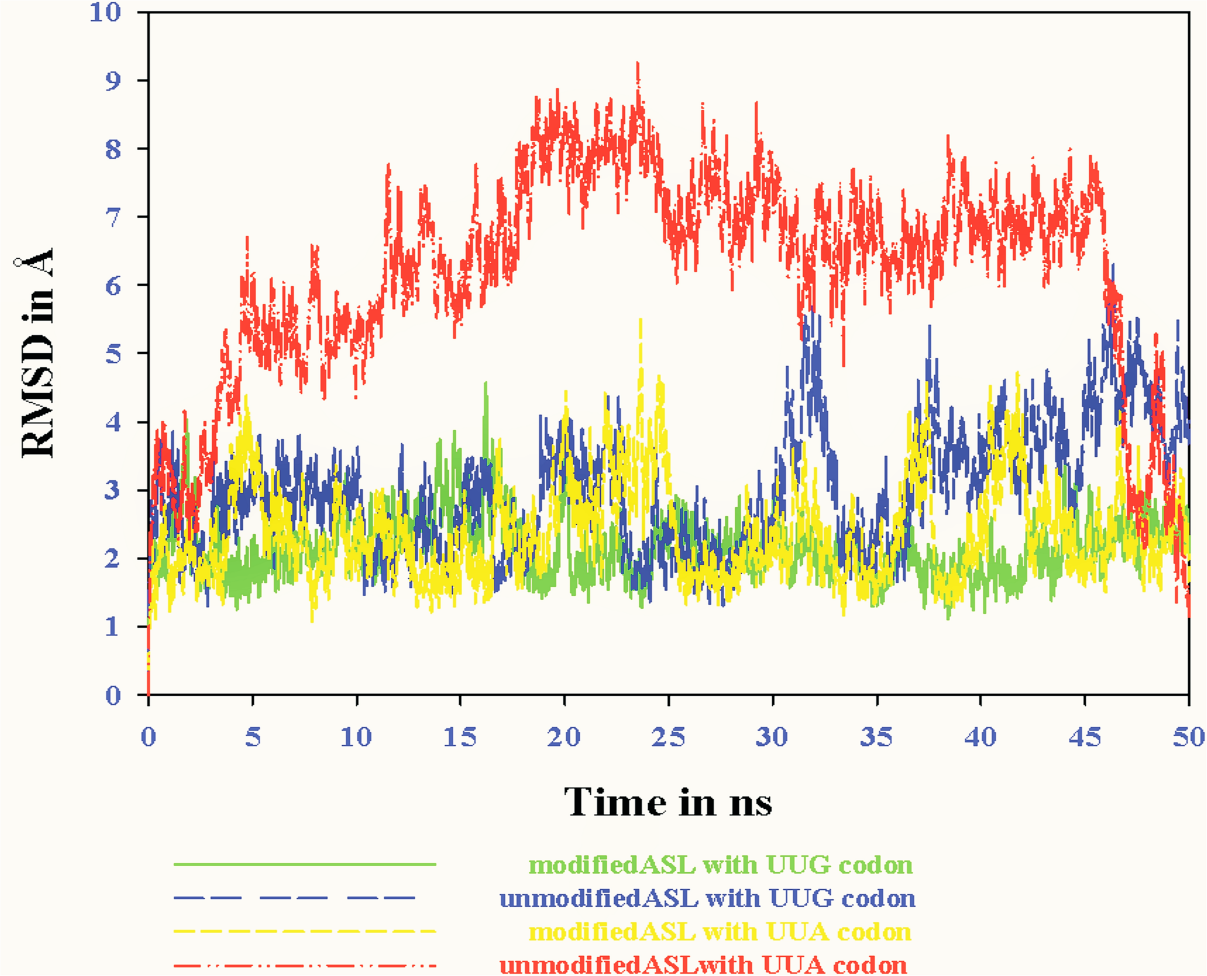
RMS deviation of ASL with the codon model sugar phosphate backbone during MD simulation in the presence and absence of modifications at the 34^th^ position.

Root mean square deviations (RMSD) of all the four models, with UUG and UUA codons, in presence and absence of τm^5^U_34_ have been elucidated in Fig 6. The average RMSD of ASL-codon with τm^5^U_34_ has been observed around 1.5 to 3.5 Å, whereas RMSD of unmodified ASL-codon (control) increases around 2 to 5 Å (Fig 6). The ASL models containing modified base τm^5^U_34_, show reduced deviations as compared to unmodified ASL models throughout MD simulation.

The RMSD of model i) has been found stable around 2Å (green color in Fig 6), whereas for model ii), it goes slightly higher (blue color in Fig 6) throughout MD simulation. In case of model ii), MD trajectory analysis revealed that unmodified uridine at 34^th^ position shows distorted binding towards G_(3)_ of UUG codon as observed in Fig 4B. The RMSD of model iii) has been observed stable around 2-3Å throughout MD simulation (yellow color in Fig 6), but slightly higher as compared to model i). On the other hand, RMSD of model iv) was found distorted as compared to models i), ii) and iii) (red color in Fig 6). Thus, RMSD analysis suggests preferred binding of anticodon loop to UUG codon in the presence of τm^5^U modification at 34^th^ position.

#### RMSF of MD simulations

**Fig 7:**
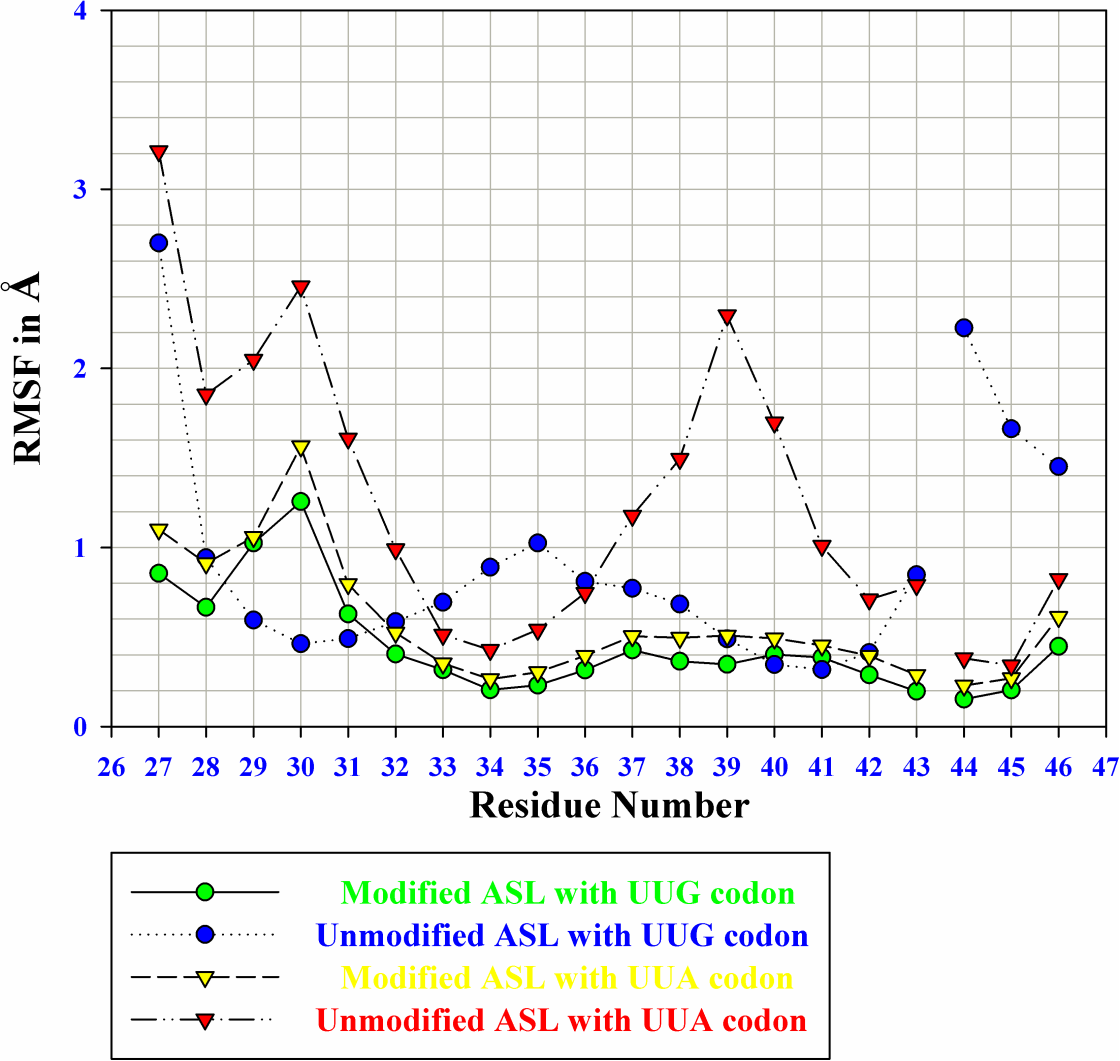
Root mean square fluctuations (RMSF) of ASL of tRNA^Leu^ residues with codons over 50 ns time scale. Residue no. 44, 45, and 46 represents codon U_1_, U_2_, and G_3_/A_3_ respectively.

Root mean square fluctuations (RMSF) of ASL tRNA^Leu^ with codons over 50 ns time scale have been shown in Fig 7. The RMSF graph of model i) shows less fluctuations (green color in Fig 7), whereas RMSF of model ii) is slightly higher (blue color in Fig 7) as compared to model i). Absence of τm^5^U_34_ directly affects the three-dimensional structure of ASL tRNA^Leu^ hence, more fluctuations were observed as compared to ASL with τm^5^U_34_. Additional fluctuations were seen in the model of ASL tRNA^Leu^ with UUA codon (Fig 7). Likewise, in model iii), RMSF graph shows more fluctuations than those observed in models i) and ii) (yellow color in Fig 7). High fluctuations were noticed in model iv) (red color in Fig 7) as compared to models i), ii) and iii). Thus, residue wise fluctuations revealed that model i) ASL with τm^5^U_34_ and UUG codon, is more stable over models ii), iii) and iv). These results also suggest the preference of τm^5^U_34_ for UUG codons over UUA.

### Analysis of MD simulation of 55S ribosomal A-site with ASL tRNA^Leu^ containing τm^5^U_34_ and UUG codon

**Fig 8:**
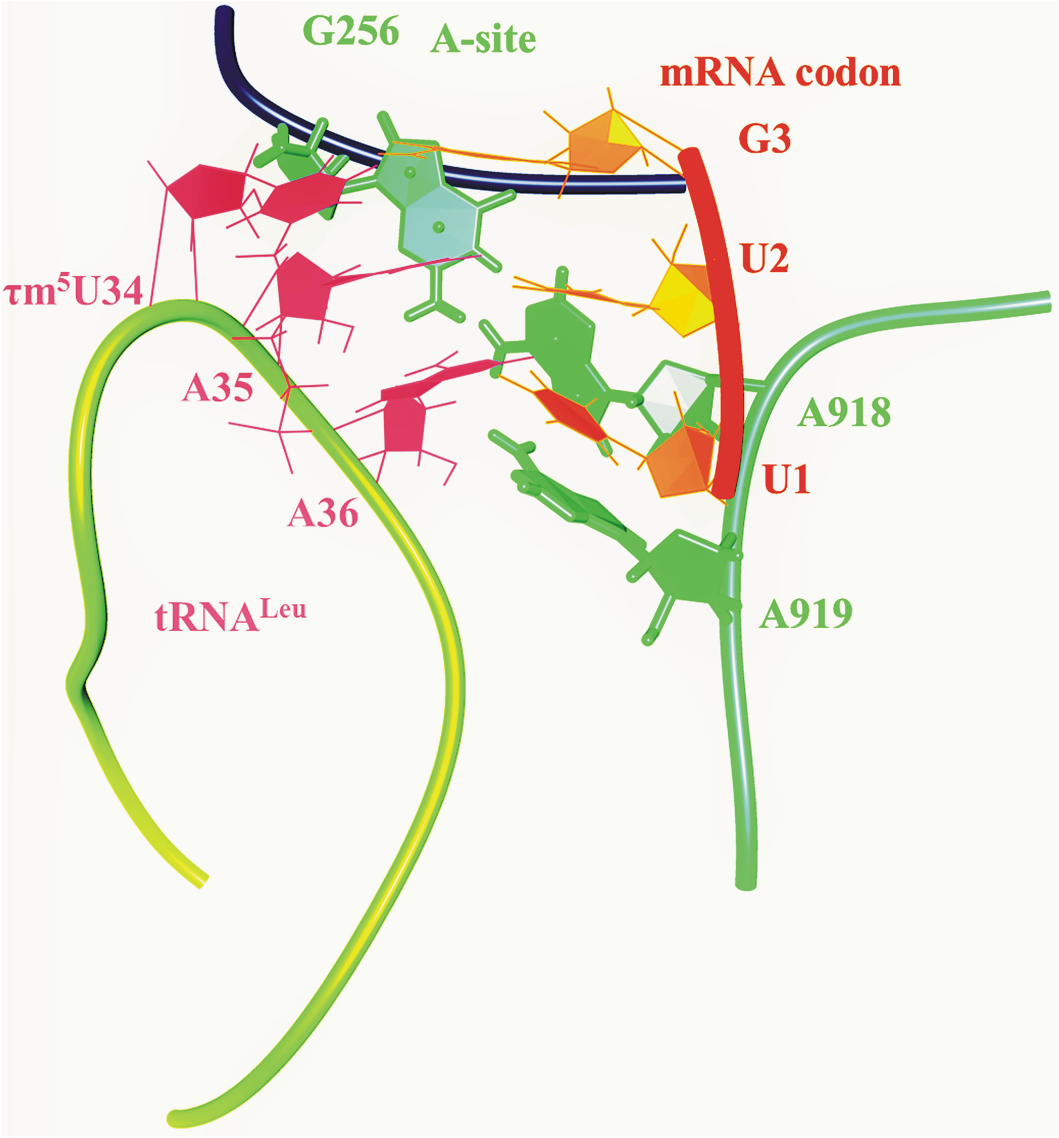
Initial structure of 55S ribosomal A-site residues (spring green color) along with ASL of tRNA^Leu^ (magenta color) with mRNA codon UUG (orange red color) considered for MD simulations.

**Fig 9:**
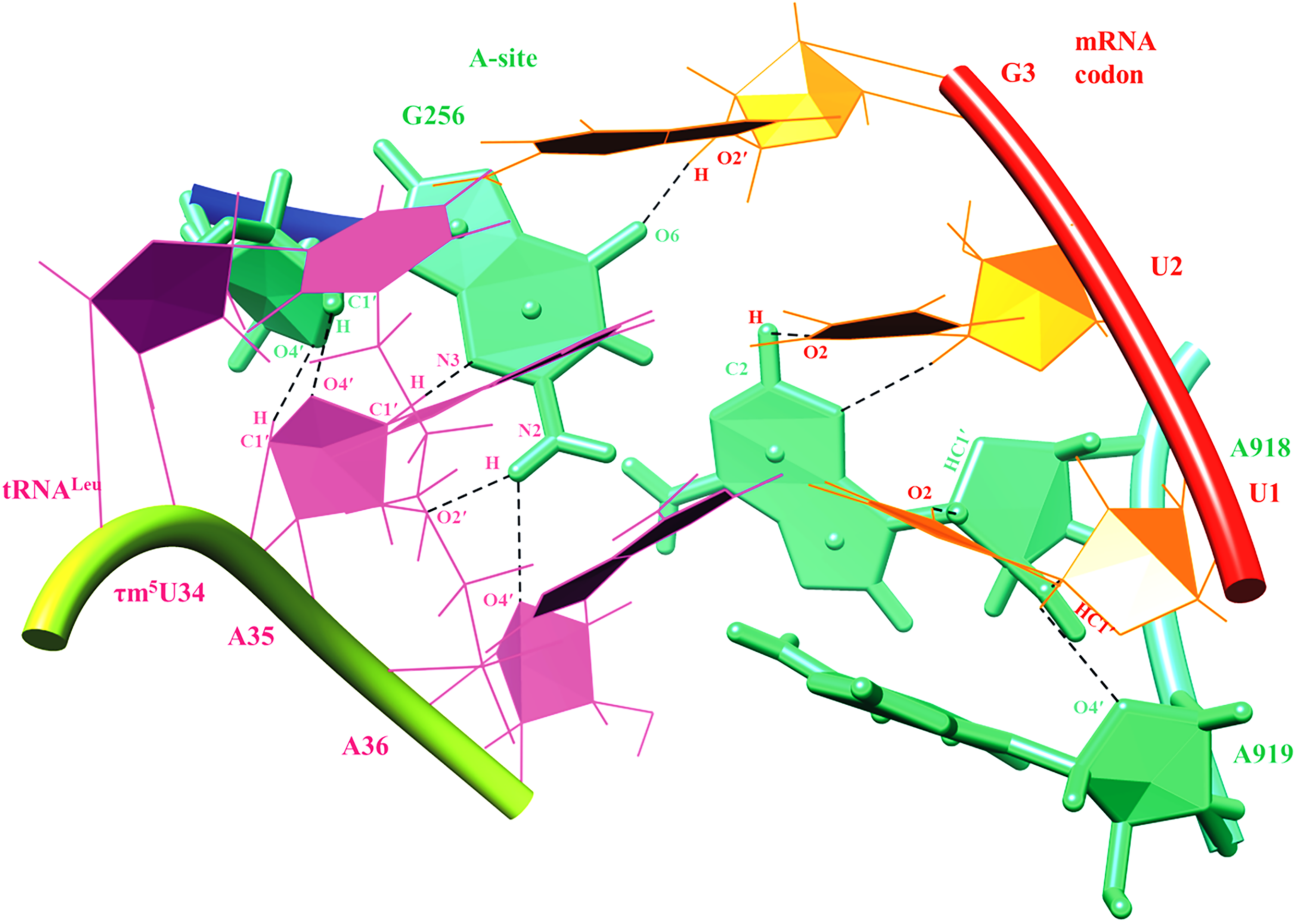
Hydrogen bonding interactions of ribosomal A-site residues (A918, A919 and G256) with ASL of tRNA^Leu^ and mRNA codon UUG.

**Fig 10:**
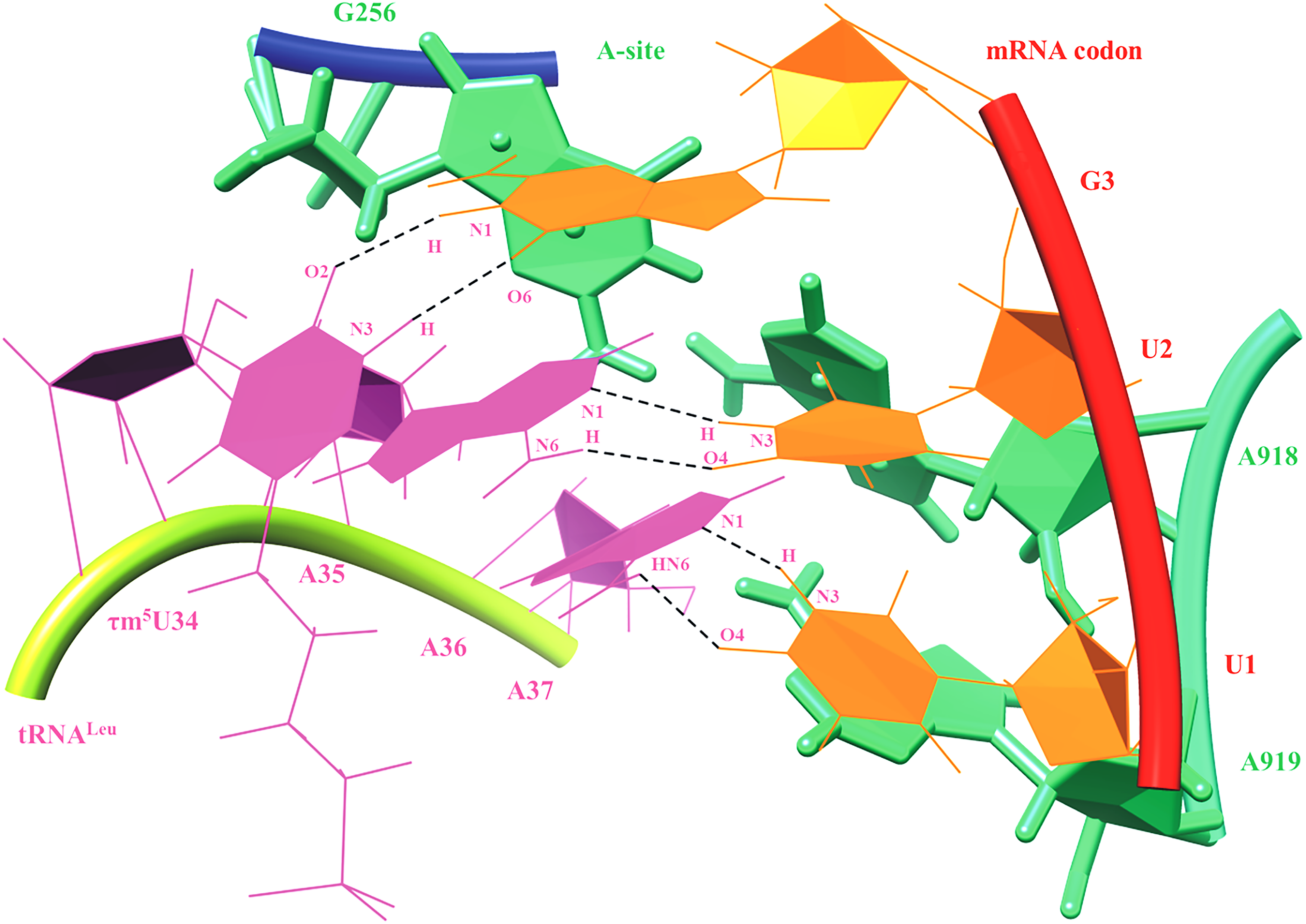
Base pairing interactions of UUG codon and ASL anticodon of tRNA^Leu^ in the context of ribosomal A-site residues.

**Fig 11:**
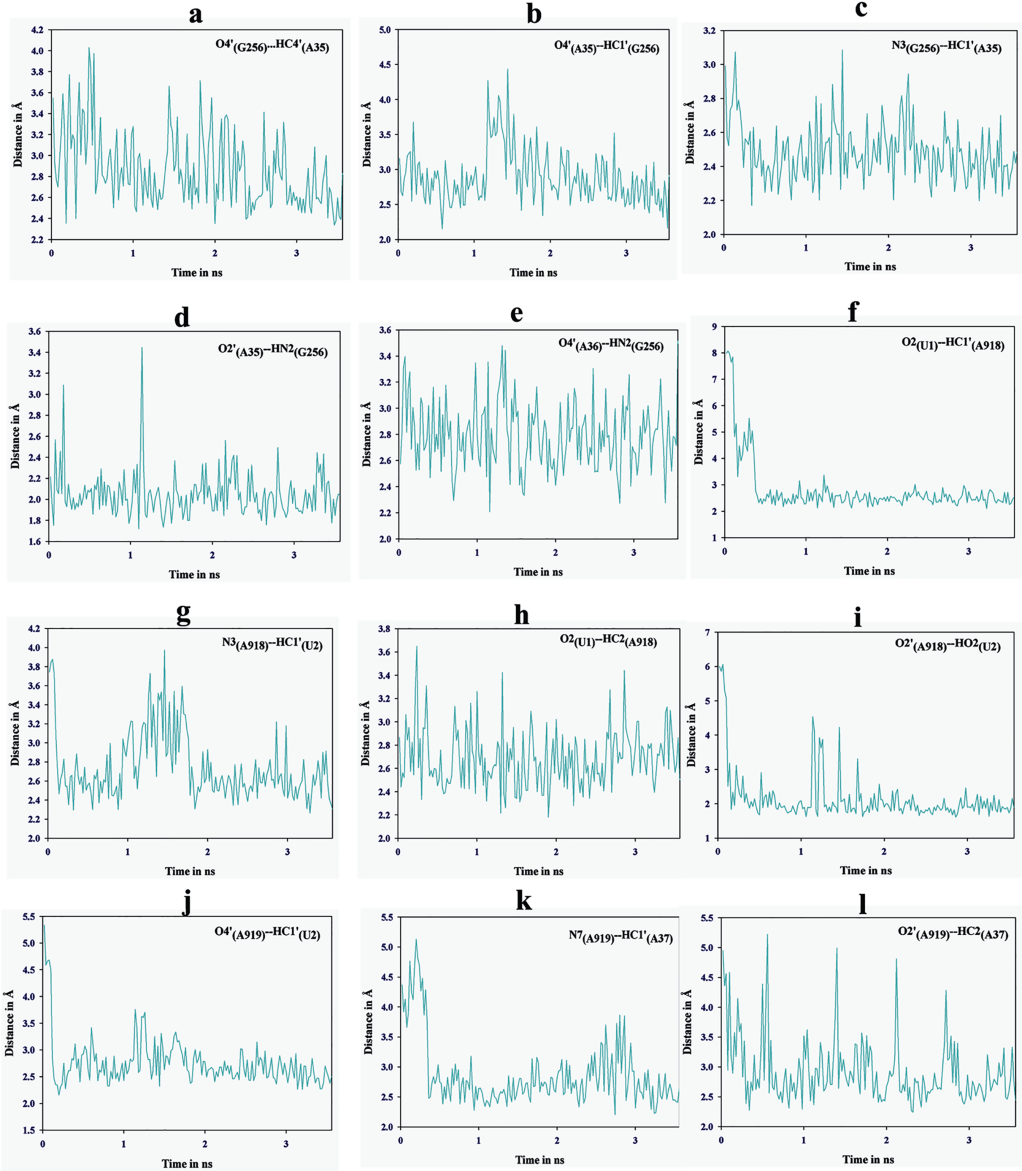
Showing fluctuations in hydrogen bonding interactions of ribosomal A-site residues (A918, A919 and G256) with ASL of tRNA^Leu^ and mRNA codon UUG.

MD simulation trajectory of ASL tRNA^Leu^ and codon UUG with the A-site of 55S mammalian mitochondrial ribosome was analyzed for hydrogen bonding interactions (Fig 8). Various interactions have been noticed which can provide stability to the codon and anticodon residues within the context of ribosomal A-site (Figs 9 and 10). Residue G256 of A-site ribosomal RNA interacts with A35 and A36 of ASL tRNA^Leu^ as well as with G3 of the mRNA codon UUG (Fig 9). Hydrogen bonding interactions between G256 and A35 are shown by atoms O4′_(G256)_…HC4′_(A35)_, O4′_(A35)_…HC1′_(G256)_, N3_(G256)_…HC1′_(A35)_, O2′_(A35)_…HN2_(G256)_ and O4′_(A36)_…HN2_(G256)_ (Fig 11). Similarly, interactions of A-site residues (A918 and A919) with ASL tRNA^Leu^ and codon UUG are listed as O2_(U1)_…HC1′_(A918),_ N3_(A918)_…HC1′_(U2)_, O2_(U1)_…HC2_(A918)_, O2′_(A918)_…HO2_(U2)_, O4′_(A919)_…HC1′_(U2)_, N7_(A919)_…HC1′_(A37)_ and O2′_(A919)_…HC2_(A37)_ (Figs 9 and 11). Thus, MD simulation results show that, ribosomal A-site residues interact with codon and anticodon bases of ASL tRNA^Leu^ to provide additional stability to the decoding complex.

## Discussions

Here, we tried to explore the structural dynamics of ASL tRNA^Leu^ in presence and absence of naturally occurring modified nucleoside, τm^5^U at 34^th^ ‘wobble’ position along with UUG/UUA codons. The stability of all models of ASL tRNA^Leu^ has been expressed in terms of hydrogen bonding interactions. The specific interactions, O4'_34_…HC(6)_34_, O5'_34_…HC(6)_34_, O(1)P_34_…HC(10)_34_, O(2)_34_…HC1'_34_, O2'_33_…HN(8)_34_ and O3'_33_…HN(8)_34_ which occur between ribose-phosphate backbone and τm^5^U_34_ side chain (Fig 2), might help to retain ‘anti’ conformation of glycosyl torsion angle and proper orientation of 5-taurinomethyl side chain of τm^5^U_34_. Hence, atoms O(4), N(3) and O(2) of τm^5^U_34_ remain free to interact with the 3^rd^ base of codon. Some hydrogen bonding interactions such as N(1)_31_…HN(4)_39_, N(7)_35_…HO2'_33_ and O(1)P_36_…HN(3)_33_ might help to conserve the U-turn feature by maintaining base stacking interactions of ASL tRNA^Leu^ (Fig 3A and 3B) as per earlier reports [7, 9]. Hydrogen bonding N(1)_31_…HN(4)_39_ between A_31_ and C_39_ (Fig 3A) could play an important role to maintain closed loop conformation of anticodon loop. This might help to enhance proper codon recognition.

The hydrogen bonding interactions observed in models i) (Fig 4A) and iii) (Fig 4C) play crucial role in maintaining proper base pairing and base stacking interactions of tRNA^Leu^ during codon recognition process. Absence of τm^5^U at 34^th^ position in models ii) (Fig 4B) and iv) (Fig 4D) does not allow to form such interactions, resulting in weak recognition of respective codons.

It has been known that during translation process, the anticodon loop of tRNA along with mRNA codons is attached to the A-site of ribosome, whereas during translocation, this A-site tRNA gets shifted to P-site [9]. In this study we have observed that the codon recognition proceeds initially by Watson-Crick type of ‘two hydrogen’ bonding interactions between O(6)_G3_…HN(3)_34_ and O(2)_34_…HN(1)_G3_ as well as O(4)_34_…HN(6)_A3_ and N(1)_A3_…HN(3)_34_ of τm^5^U_34_ with G_3_/A_3_ of codons respectively (Fig 5A and 5E). Later, out of these two hydrogen bonds, a single hydrogen bond depicted by O(6)_G3_…HN(3)_34_ (Fig 5B) and N(1)_34_…HN(3)_A3_ (Fig 5D) may further stabilize the codon-anticodon complex. This single hydrogen bond might play a significant role during protein biosynthesis process.

RMSD results revealed the stability of ASL tRNA^Leu^ with UUG and UUA codons. The steadiness of RMSD graph of model i) (Fig 6) depends upon proper base stacking and hydrogen bonding interactions due to the presence of τm^5^U at 34^th^ position. It has been proven that the lack of modification at 34^th^ ‘wobble’ position hampers proper codon anticodon base pairing [35]. The deviations observed in RMSD graph of model ii) might be due to the lack of τm^5^U modification at 34^th^ ‘wobble’ position. Reduced decoding of UUA codon was reported in case of MELAS mutant tRNA^Leu^ [35]. This is also evident from the RMSD of model iii), which is slightly higher as compared to model i). The distortions observed in RMSD graph of model iv), might be because of two factors, first the absence of τm^5^U modification at 34^th^ position and the other is reduced UUA decoding as reported in earlier study [35]. This indicates that the modification τm^5^U at 34^th^ position is crucial for the stability of ASL of human mt tRNA^Leu^.

The RMSF graph showing residue wise fluctuations of ASL bases with UUG/UUA codons is depicted in Fig 7. RMSF values of models i) and iii), show minor fluctuations than those of models ii) and iv). This indicates that the presence of τm^5^U_34_ enhances stacking interactions among the bases of ASL tRNA^Leu^, thus showing lesser fluctuations. These fluctuations are the outcome of presence or absence of modified base τm^5^U at 34^th^ position of ASL tRNA^Leu^ which directly contributes to tRNA conformation.

55S mammalian mitochondrial ribosomal A-site residue, G256 interacts with ribose-phosphate backbone of A35, A36 of ASL tRNA^Leu^ and G_3_ of mRNA codon, providing additional stability to the codon-anticodon complex (Figs 9–11). Ribosomal residues, A918 and A919 interact with U_1_ and U_2_ of codons. These interactions could be useful to maintain the codon-anticodon decoding complex in the A-site of ribosome.

## Conclusion

Molecular dynamics simulation results of tRNA^Leu^ containing τm^5^U_34_ with UUG/UUA codons, show proper base stacking and base pairing interactions, whereas in absence of τm^5^U_34_, distorted interactions were found, suggesting the significant role of τm^5^U_34_. Ribose-phosphate backbone interactions and ‘anti’ conformation of glycosyl torsion angle of τm^5^U_34_ play crucial role in codon recognition by making Watson–Crick base pairing sites, O(4), N(3) and O(2) of τm^5^U_34_ freely available to interact with 3^rd^ base of codons. MD simulation trajectories showed alternating presence of ‘wobble’ and ‘novel’ single hydrogen bonding interactions between τm^5^U_34_:G_3_/A_3_ base pairs. This ‘novel’ single hydrogen bond might play a significant role during the protein biosynthesis process. However, further long duration MD simulations of tRNA^Leu^, mRNA and whole ribosomal complex are necessary to study translocation of the tRNA from A-site to P-site.

The 55S mammalian mitochondrial A-site residues, G256, A918 and A919 provide proper support for the decoding process. These results show increased decoding efficiency of human mt tRNA^Leu^ for UUG codon over UUA in presence of modified base, τm^5^U at wobble 34^th^ position. Thus, this study helps to understand the structural basis of proper codon recognition by τm^5^U_34_, which is hampered in case of MELAS.
